# Continuing evolution of highly pathogenic H5N1 viruses in Bangladeshi live poultry markets

**DOI:** 10.1080/22221751.2019.1605845

**Published:** 2019-04-24

**Authors:** Subrata Barman, Jasmine C. M. Turner, M. Kamrul Hasan, Sharmin Akhtar, Rabeh El-Shesheny, John Franks, David Walker, Patrick Seiler, Kimberly Friedman, Lisa Kercher, Trushar Jeevan, Pamela McKenzie, Richard J Webby, Robert G Webster, Mohammed M Feeroz

**Affiliations:** a Department of Infectious Diseases, St. Jude Children’s Research Hospital, Memphis, TN, USA; b Department of Zoology, Jahangirnagar University, Dhaka, Bangladesh; c Center of Scientific Excellence for Influenza Viruses, National Research Centre, Giza, Egypt

**Keywords:** Bangladesh, live poultry market, avian influenza A virus, highly pathogenic H5N1 viruses, H9N2 viruses, domestic ducks, reassortment, surveillance

## Abstract

Since November 2008, we have conducted active avian influenza surveillance in Bangladesh. Clades 2.2.2, 2.3.4.2, and 2.3.2.1a of highly pathogenic avian influenza H5N1 viruses have all been identified in Bangladeshi live poultry markets (LPMs), although, since the end of 2014, H5N1 viruses have been exclusively from clade 2.3.2.1a. In June 2015, a new reassortant H5N1 virus (H5N1-R1) from clade 2.3.2.1a was identified, containing haemagglutinin, neuraminidase, and matrix genes of H5N1 viruses circulating in Bangladesh since 2011, plus five other genes of Eurasian-lineage low pathogenic avian influenza A (LPAI) viruses. Here we report the status of circulating avian influenza A viruses in Bangladeshi LPMs from March 2016 to January 2018. Until April 2017, H5N1 viruses exclusively belonged to H5N1-R1 clade 2.3.2.1a. However, in May 2017, we identified another reassortant H5N1 (H5N1-R2), also of clade 2.3.2.1a, wherein the PA gene segment of H5N1-R1 was replaced by that of another Eurasian-lineage LPAI virus related to A/duck/Bangladesh/30828/2016 (H3N8), detected in Bangladeshi LPM in September 2016. Currently, both reassortant H5N1-R1 and H5N1-R2 co-circulate in Bangladeshi LPMs. Furthermore, some LPAI viruses isolated from LPMs during 2016–2017 were closely related to those from ducks in free-range farms and wild birds in Tanguar haor, a wetland region of Bangladesh where ducks have frequent contact with migratory birds. These data support a hypothesis where Tanguar haor-like ecosystems provide a mechanism for movement of LPAI viruses to LPMs where reassortment with poultry viruses occurs adding to the diversity of viruses at this human-animal interface.

## Introduction

In 1997, highly pathogenic avian influenza (HPAI) H5N1 viruses emerged as a human pathogen in Hong Kong Special Administrative Region, China [1]. Since 2003, H5N1 viruses have spread from East Asia, across Eurasia, to as far as England and West Africa. The spread of HPAI H5N1 viruses has led to dramatic economic losses to poultry industries. Zoonotic H5N1 viruses are also a major public health concern. Since the reemergence of H5N1 viruses in 2003, 860 laboratory-confirmed cases of human infection, including at least 454 deaths, have been reported to the World Health Organization (WHO) [2].

In Bangladesh, HPAI H5N1 viruses were first identified in February 2007 [3]. These viruses have co-circulated alongside low pathogenic avian influenza (LPAI) viruses, predominantly of the H9N2 subtype. So far three clades, 2.2.2, 2.3.4.2, and 2.3.2.1a, of H5N1 have been identified in Bangladesh [4–6]. By the end of 2014, H5N1 viruses in Bangladesh exclusively belonged to clade 2.3.2.1a [5].

Bangladesh is located at the overlap of two major flyways for migratory birds – the Central Asian and East Asian-Australian flyways [7–10]. The northeastern Sunamganj district of Bangladesh consists of marshy seasonal wetlands, termed haors, where floodplains and tributaries receive surface runoff to form seasonal lakes. Haors provide abundant aquatic vegetation for migratory waterfowl to overwinter from across Europe and Central Asia [11,12]. Commercially raised ducks in these haors commonly scavenge for food during the day, thereby making frequent contact with migratory waterfowl. Hence, resident poultry are at high risk of acquiring LPAI infections and often contribute to the dispersal of the vast gene pool of LPAI viruses. Although several LPAI virus subtypes (primarily H9N2) co-circulate with H5N1 viruses in Bangladeshi live poultry markets (LPMs) [11], reassortment between HPAI H5N1 and LPAI viruses is somewhat rare. Reassortant H5N1 viruses with the matrix (M) gene from H9N2 viruses of Chinese lineage [4] or the polymerase basic 1 (PB1) gene from Bangladeshi H9N2 viruses [5,13–15] have been isolated from LPMs in Bangladesh, but they failed to establish and quickly disappeared. However, in June 2015 we identified a reassortant H5N1 virus of clade 2.3.2.1a (H5N1-R1), containing the haemagglutinin (HA), neuraminidase (NA), and matrix (M) genes of H5N1 viruses circulating in Bangladesh since 2011 and five other genes of Eurasian-lineage LPAI viruses. H5N1-R1 quickly established itself as the predominant H5N1 virus in Bangladeshi LPMs [15].

Reassortant H5N1-R1 of clade 2.3.2.1a was circulating as the lone genotype until April 2017 in Bangladeshi LPMs. Then in May 2017, another reassortant H5N1 virus of clade 2.3.2.1a (H5N1-R2) appeared in which the H5N1-R1 PA gene segment was replaced by that of another Eurasian-lineage LPAI virus. Since then, both reassortant H5N1-R1 and H5N1-R2 viruses have co-circulated in Bangladeshi LPMs. We also demonstrate that some LPAI viruses isolated from LPMs during 2016–2017 were closely related to those isolated in 2015 from Tanguar haor, a wetland region of Bangladesh where farm-raised ducks have frequent contact with migratory birds.

## Materials and methods

### Sample collection

Oropharyngeal, cloacal, and water samples were collected (March 2016 –January 2018) from poultry (chickens, ducks, and quail) and poultry cages at six LPMs in or near Dhaka, Bangladesh. Samples were collected as previously described [4,5,15,16]. Samples were stored on wet ice blocks (approximately 4°C) in the field and moved to liquid nitrogen storage within one week. After receiving air carrier approval, every two months samples were shipped with collection details to high-security biosafety level 3+ facilities at St. Jude for analysis.

### Sample screening and virus isolation

All samples were screened by real-time reverse transcription PCR with universal M gene- and then with H5 HA-specific primers, as previously described [16,21].

FluA-positive samples were propagated in 10-day-old embryonated chicken eggs for virus isolation. We previously observed that non-H5, FluA-positive samples from domestic chickens and quail yield mostly H9N2 isolates and other LPAI isolates are rare. Therefore, we cultured approximately 10% of selected non-H5, FluA-positive samples from chickens and quail, which represented each sampling period and location. However, for ducks, all FluA-positive samples, whether H5 positive or negative, were inoculated in eggs.

### Genome sequencing and phylogenetic analysis

Viral RNA was extracted using the RNeasy Mini Kit (Qiagen), and cDNA was synthesized using the Superscript RT III Reverse Transcriptase Kit (Invitrogen). PCR of influenza A virus gene segments was performed using primers described elsewhere [22]. PCR products were extracted from 1.2% agarose gel and purified using a DNA purification kit (GE Healthcare). Libraries were prepared using the Nextera XT DNA Library Prep Kit (Illumina), according to the manufacturer’s protocol. Libraries were sequenced using the MiSeq System (Illumina). Sequencing reads were then de-multiplexed, quality trimmed, and filtered using CLC Genomics Workbench 7 (CLC Bio). A total of 1298 gene segment sequences obtained from 171 isolates in this study were deposited to the Influenza Research Database and are available under GenBank accession numbers MG042083-MG042468, MG957503-MG957633, MH135325-MH135700, and MH791537-MH791941.

For phylogenetic analyses, sequences other than those found in this study were retrieved from the National Center for Biotechnology Information Influenza Virus Sequence Database [23] and the EpiFlu database of the Global Initiative on Sharing All Influenza Data [24]. Sequences were then aligned, and ends were trimmed to equal lengths with BioEdit sequence alignment editor software (version 7.0.5) [25]. Similar sequences, including sequences obtained in this study, were removed and phylogenetic relationships were inferred by the neighbor-joining method from 500 bootstrap values; topology was confirmed by the maximum likelihood method [26]; and evolutionary analyses were conducted with the MEGA 7 software [27].

### Haemagglutination inhibition assays

Haemagglutination inhibition (HI) assays were performed as previously described [4,15]. The panel of antisera used in HI assays included representatives from currently circulating genetic lineages of H5N1 clade 2.3.2.1 and H9N2 G1 and Y280/G9 in Asia, including Bangladesh. For HI assays, 0.5% chicken red blood cells were used.

## Results

### Avian influenza A surveillance in Bangladeshi LPMs

**Figure 1.**
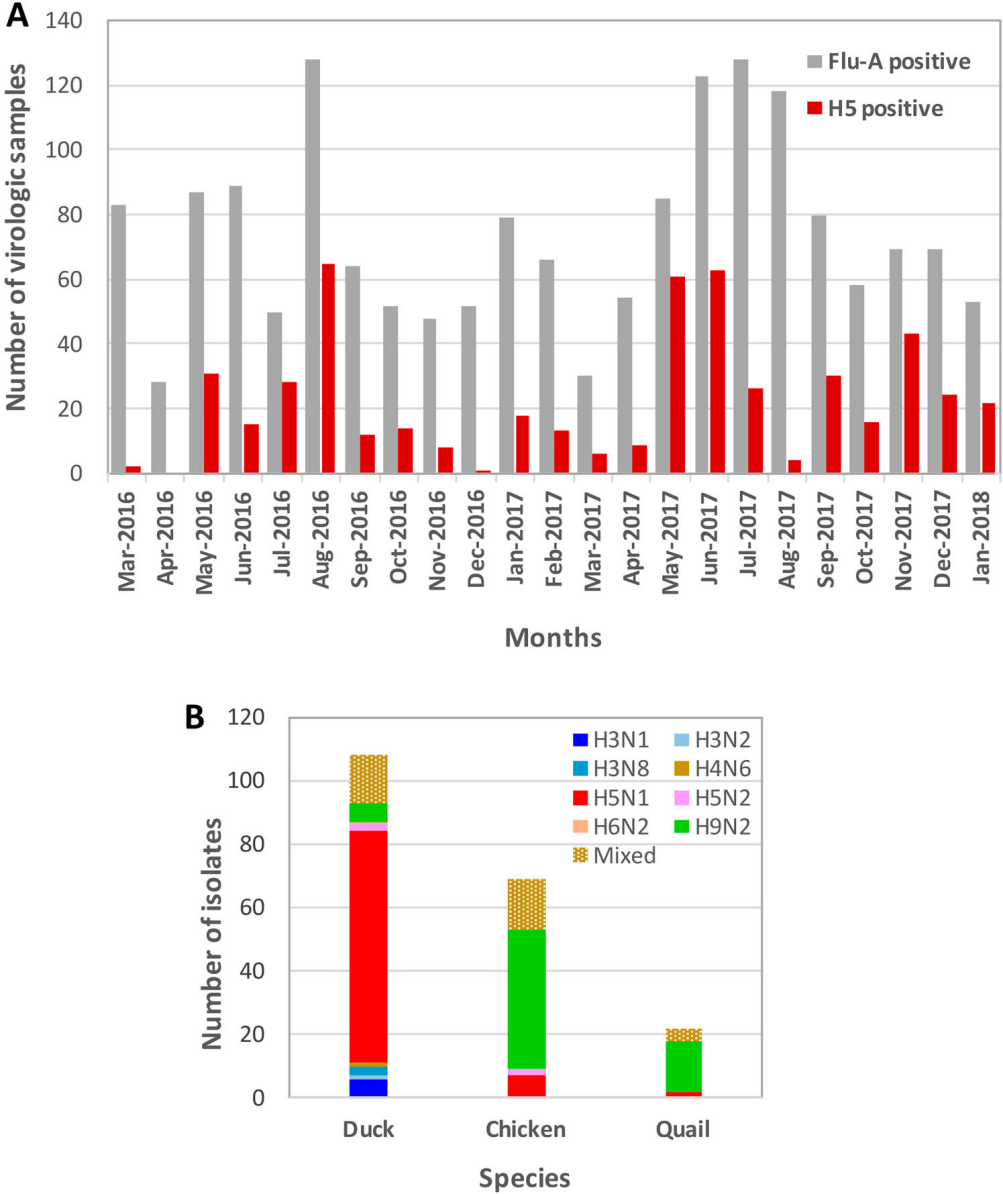
Avian influenza surveillance in Bangladesh live poultry markets (March 2016 – January 2018). Each month, 175 virologic samples were collected and screened. **(A)** FluA (grey) and H5 (red) positive samples were determined by M and H5-HA gene-specific real-time PCR, respectively and plotted against the months samples were collected. **(B)** Viruses isolated from ducks, chickens, and quail in LPMs. All FluA-positive duck samples and H5-positive samples from any species were inoculated in eggs. However, only about 10% of H5 negative but FluA-positive chicken and quail samples were inoculated in eggs. Mixed viruses were predominantly H5N1/H9N2, while other combinations of mixed subtypes were isolated less frequently.

Monthly collection (March 2016 –January 2018) of 175 virologic samples (oropharyngeal, cloacal, and water) from poultry (chickens, ducks, and quail) and poultry cages at six LPMs in and around Dhaka, Bangladesh. The prevalence of FluA-positive samples greatly varied from 16% (28 of 175 samples) in April 2016 to as high as 73% (128 of 175 samples) in July 2017, with an average of 43% (Figure 1(A)). Average prevalence of FluA-positive samples for ducks, chickens, and quails was 40%, 39%, and 55%, respectively, which is consistent with our previous reports [4,5,15,16]. There was no specific pattern of seasonality for the prevalence of FluA-positive samples. However, in August 2016 (73%) and again in June (70%), July (73%), and August (66%) 2017, the prevalence of FluA-positive samples was much higher than average (43%). The prevalence of H5-positive samples varied greatly, from 1.1% (2 of 175 samples) and 0.6% (1 of 175 samples) in March and December 2016, respectively, to as high as 37% (65 of 175 samples), 35% (61 of 175 samples), and 36% (63 of 175 samples) in August 2016, May 2017, and June 2017, respectively, with an average of 13% (511 of 4025 total samples). In the month of April 2016, when the lowest number of FluA-positive samples was detected, no H5-positive samples were identified (Figure 1(A)).

We previously observed that non-H5, FluA-positive samples from domestic chickens and quail are mostly H9N2 isolates and other LPAI isolates are rare. Therefore, we cultured approximately 10% of selected non-H5, FluA-positive samples from chickens and quail, which represented each sampling period and location. However, for ducks, all FluA-positive samples, whether H5 positive or negative, were inoculated in eggs. We isolated several AIV subtypes from different species of birds in LPMs (Figure 1(B)) and found H5N1 viruses predominantly in duck samples. LPAI viruses isolated from Bangladeshi LPMs were mostly H9N2 and predominantly isolated from chickens and quails. LPAI viruses other than H9N2 were rare and almost exclusively isolated from ducks. Viruses of H5N1/H9N2 mixed subtype were frequently isolated, while other combinations of mixed subtypes were isolated less frequently.

### HA and NA phylogeny of LPAI viruses

Phylogenetic analysis revealed that HA gene segments of H3, H4, and H6 viruses isolated from LPMs were all of Eurasian lineage (Figure 2(A–C)). The HA genes of H3 viruses (Figure 2(A)) formed two distinct genetic groups. One group containing three H3N8 viruses and one H3N2 virus that clustered together with viruses from Mongolia and Russia (Chany). The second group consisted Bangladeshi H3N1 and H3N2 viruses together with viruses from China, Mongolia, Russia (Chany), and Viet Nam, and, most importantly, with viruses such as A/duck/Bangladesh/26918/2015(H3N6) isolated from Tanguar haor (Bangladesh) during 2015 [15]. The H4 HAs (Figure 2(B)) were closely related to H4N6 viruses isolated from Mongolia in 2015 and Bangladesh in 2010. The HA of H6N2 virus clustered with those of H6N1 or H6N2 viruses isolated from Bangladesh, China, and Siberia (Figure 2(C)). Phylogenetic analysis of HA and NA genes of H9N2 viruses indicated homology with those of H9N2 viruses isolated from Bangladeshi LPMs during 2011–2016 (Figure S1A and S1B). The NA genes of N1, N2, N6, and N8 LPAI viruses were all of Eurasian lineage (Figure S1B and S2A–C). Figure 2.Phylogenetic relationship of haemagglutinin (HA) genes of (**A**) H3, (**B**) H4, and (**C**) H6 LPAI viruses isolated in Bangladeshi LPMs. Viruses identified during the current surveillance period are depicted in blue. Tree is rooted to midpoint. Bootstrap values ≥70% are indicated on branches.

**Figure:**
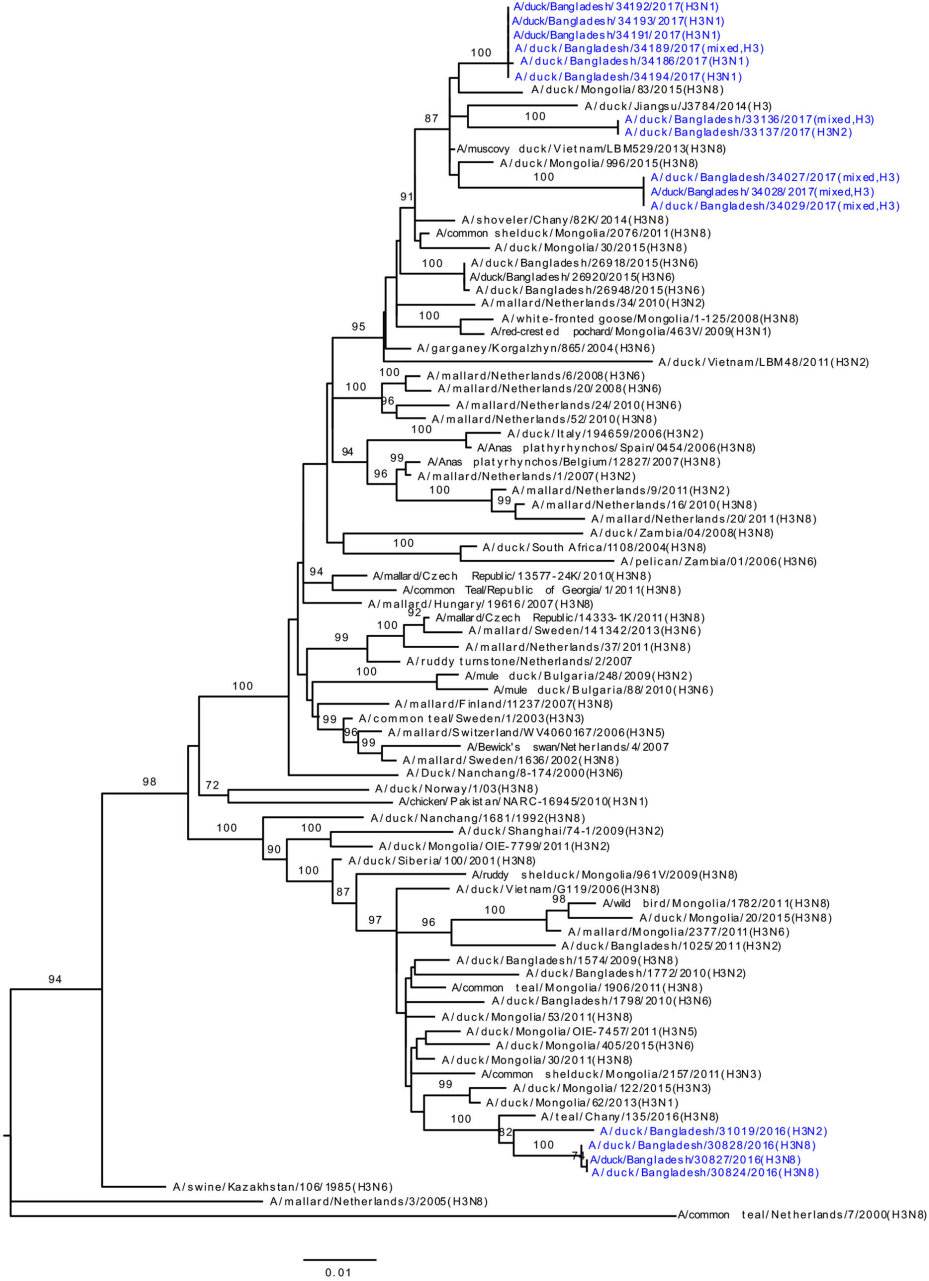

**Figure:**
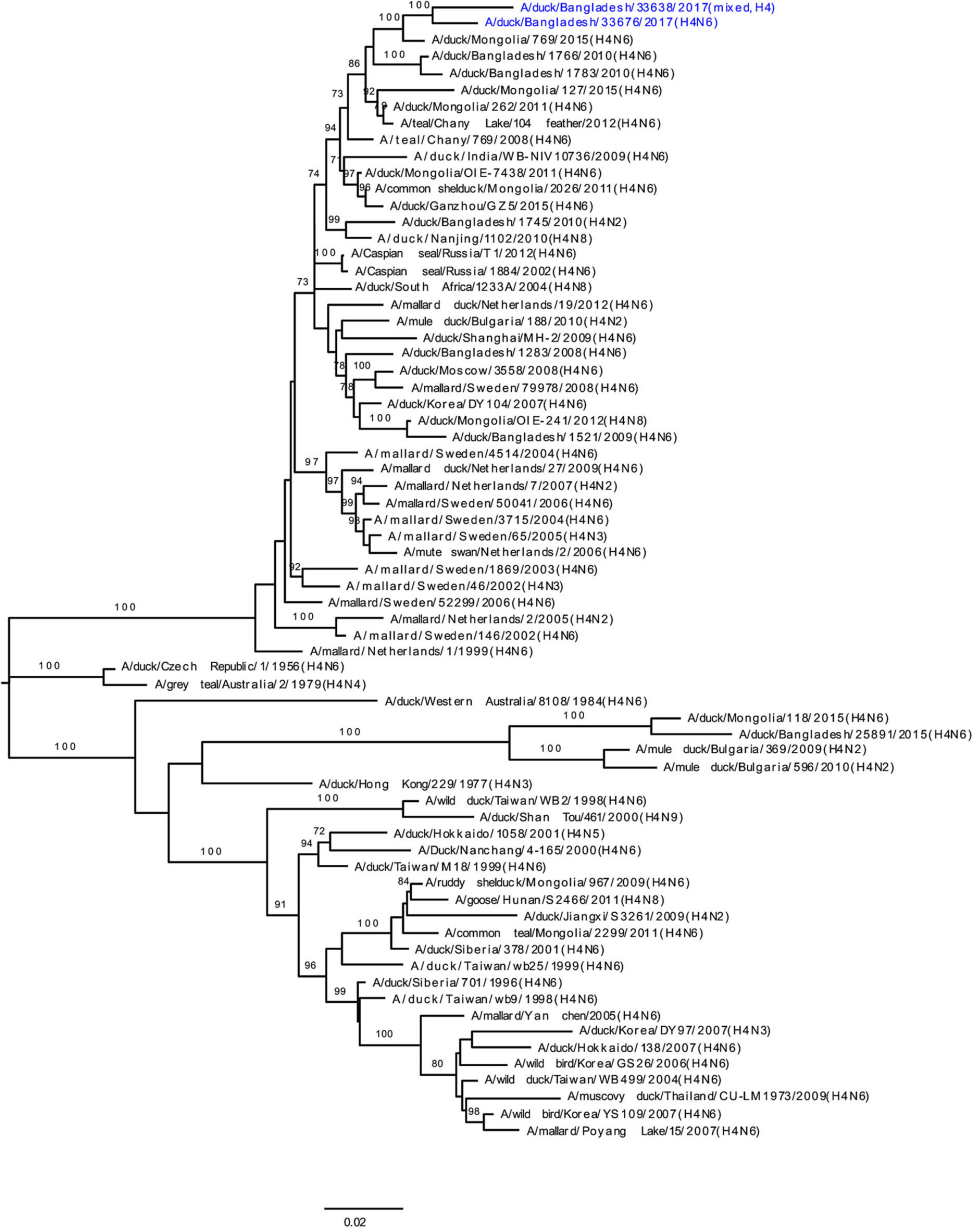

**Figure:**
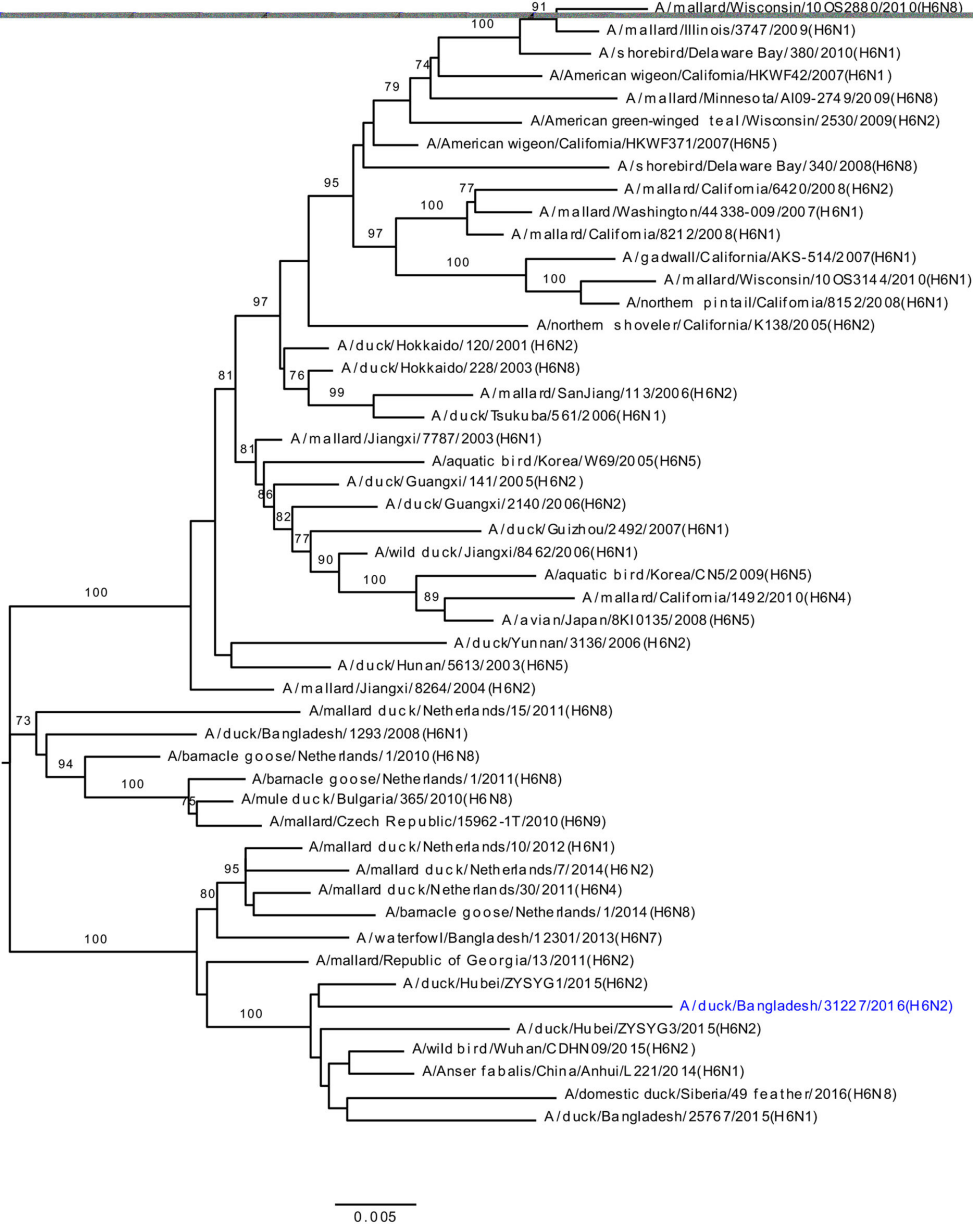

### HA and NA phylogeny of H5N1 viruses

**Figure 3.**
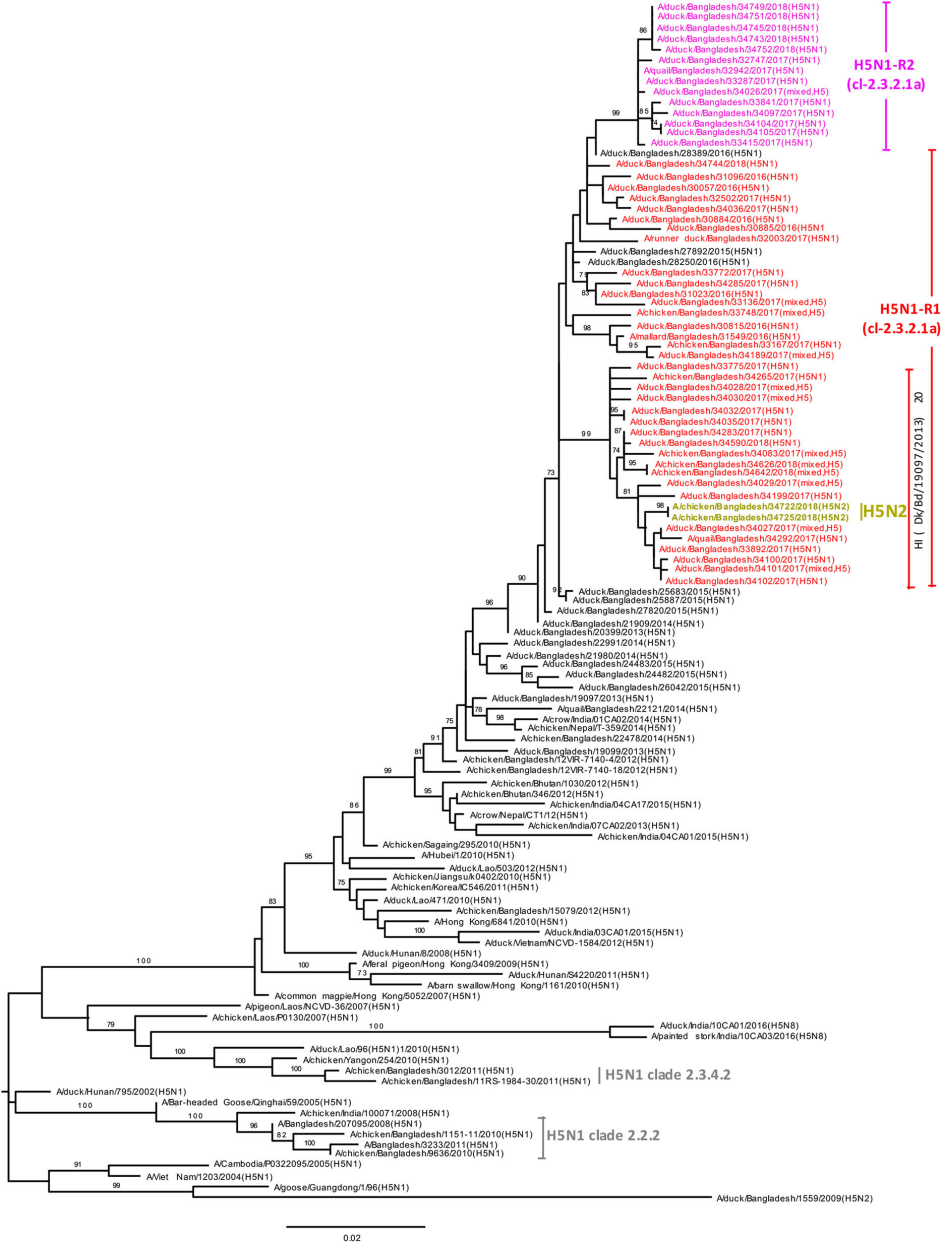
Phylogenetic relationship of haemagglutinin (HA) genes of HPAI H5N1 viruses isolated in Bangladeshi LPMs. The complete coding region of HA1 was used. Viruses identified during the surveillance period are colour coded (red, H5N1-R1; purple, H5N1-R2; and yellow-green, H5N2 viruses). *, Post-infection ferret antisera used for HI assays. Note that the homologous HI titre for A/Bangladesh/19097/2013(H5N1) was 320, and HI titres for recent H5N1-R1 (other than indicated) and H5N1-R2 viruses were between 80 and 160. Dk, duck; Bd, Bangladesh. •, HPAI H5N1 viruses isolated from humans in Bangladesh. Tree is rooted to midpoint. Bootstrap values ≥70% are indicated on branches.

According to the H5N1 HA phylogenetic tree, the HA genes of all Bangladeshi H5N1 viruses isolated during the surveillance period formed a monophyletic cluster within clade 2.3.2.1a H5N1 viruses that shared common ancestry with viruses from Bangladesh, Bhutan, Nepal, and India (Figure 3). Furthermore, the HA genes of 14 recently isolated H5N1-R2 viruses (purple; Figure 3) clustered as a distinct homologous subgroup that shared a common ancestor with A/duck/Bangladesh/28389/2016(H5N1). HA genes of two H5N2 viruses (yellow-green, Figure 3) isolated during January 2018 clustered with H5N1 of clade 2.3.2.1a viruses isolated around the same time and were distinct from the 14 H5N1-R2 HAs. The NA genes of H5N1 viruses also clustered with those of H5N1 viruses circulating in Bangladesh since 2013 (Figure S3). Similar to HA genes, NA genes of the same 14 recently isolated viruses formed a distinct homologous subgroup, sharing common ancestry with A/duck/Bangladesh/28389/2016(H5N1) (purple; Figure S3).

### Antigenic analysis in haemagglutination inhibition (HI) assays

We used WHO reference antisera and post-infection ferret antisera against viruses previously isolated from Bangladesh for HI assays. On the basis of phylogenetic analysis of HA genes, H5N1 and H9N2 viruses were selected for HI assays. A group of H5N1 viruses had reduced reactivity to ferret antisera against A/duck/Bangladesh/19097/2013(H5N1), with HI titres ≤20 compared with a homologous HI titre of 320 (Table S1), indicating considerable antigenic drift. Interestingly, the phylogenetic tree revealed that all HA genes of these poorly reactive viruses clustered together with signature amino acid changes including position 154 and 156 (Figure 3 and Table S2).

There were no overall changes in the antigenicity of H9N2 viruses isolated during the surveillance period (Table S3), compared with previously isolated viruses from Bangladeshi LPMs [4,5,15]. Most H9N2 viruses isolated from quail had distinctive HI patterns, consistent with our previous observations [15,17]. However, the antigenicity of the two H9N2 viruses isolated from quail (A/quail/Bangladesh/32525/2017 and A/quail/Bangladesh/32935/2017) was similar to that of chicken H9N2 isolates, indicating that this is not universal case. Phylogenetic analysis also revealed that HA genes of these two viruses clustered with HA genes from chicken isolates (Figure S1A) indicating fresh introduction of chicken H9N2 to quail.

### Phylogeny of internal genes

**Figure 4.**
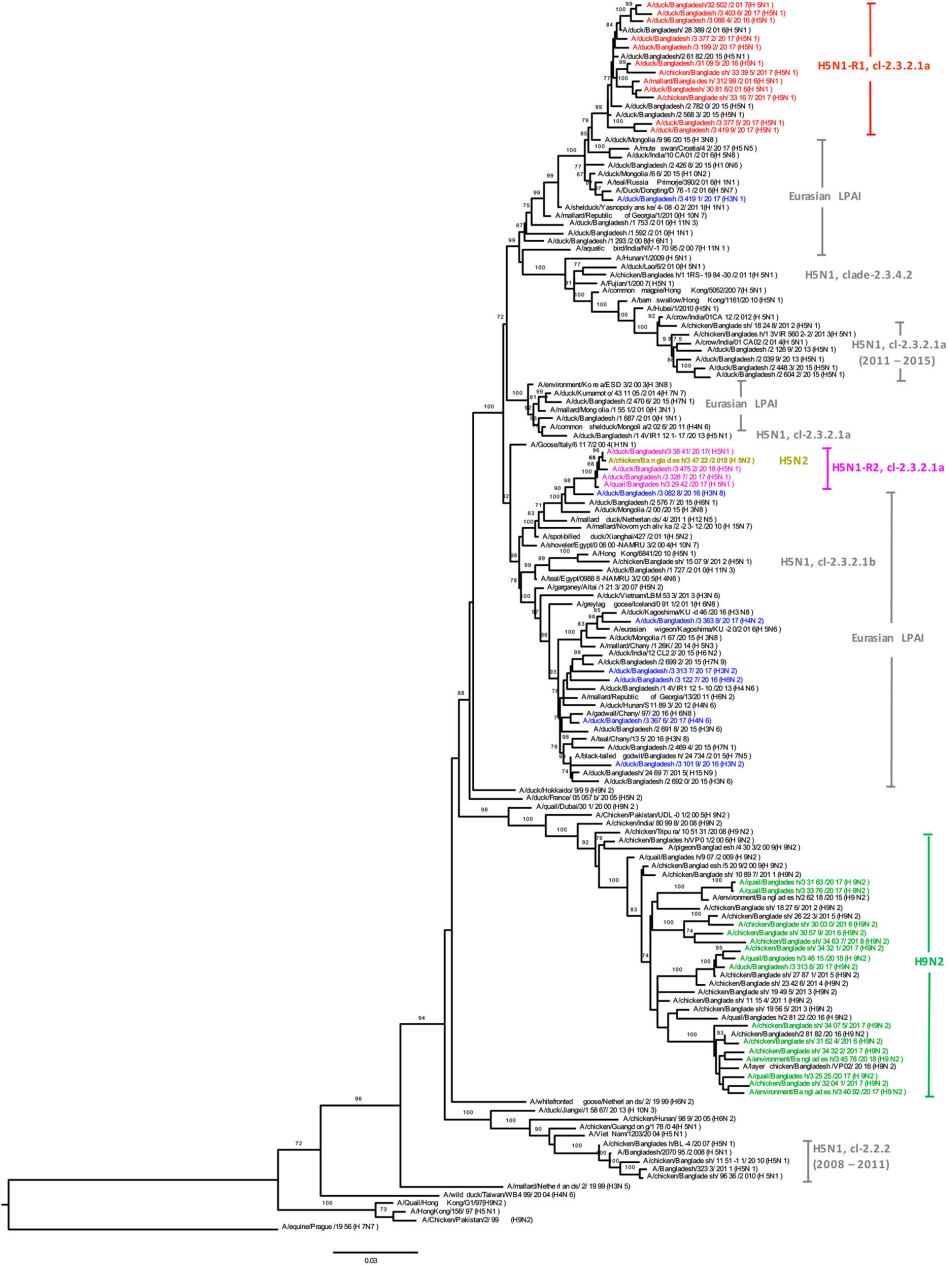
Phylogenetic relationship of PA genes of viruses isolated in Bangladeshi LPMs. For this, 1896 nt (from positions 85 nt through 1980 nt [positive sense]) were used. Viruses identified during the surveillance period are colour coded (red, H5N1-R1; purple, H5N1-R2; and yellow-green, H5N2; green, H9N2; and blue, LPAI [non-H9N2] viruses). •, HPAI H5N1 viruses isolated from humans in Bangladesh. ♦, Eurasian LPAI viruses isolated from Tanguar haor in 2015. Tree is rooted to the PA sequence of A/equine/Prague/1/1956(H7N7). Bootstrap values ≥ 70% are indicated on branches.

We determined the phylogeny of all six internal genes of viruses isolated from Bangladeshi LPMs. Phylogenetic analysis of PA genes segregated the viruses into three major clusters: one for H9N2 viruses, and two separate clusters, containing both HPAI H5N1 and non-H9N2 LPAI viruses (Figure 4). The PA genes of LPAI viruses were all of Eurasian lineage and formed two distinct clusters (blue; Figure 4). The PA gene of A/duck/Bangladesh/34191/2017(H3N1) clustered with those of LPAI viruses isolated from Bangladesh, Mongolia, Russia, and China from 2015–2016 (Figure 4). The PA genes of the other six LPAI viruses (two H3N2, one H3N8, one H4N2, one H4N6, and one H6N2) were all of Eurasian lineage (Figure 4). Interestingly, the PA genes of A/duck/Bangladesh/31019/2016(H3N2) clustered with those of viruses isolated from Tanguar haor in 2015 (Figure 4) [15]. These findings indicate probable movement of LPAI viruses from the Tanguar haor-like ecosystem to LPMs, although it is not possible to exclude a common source of virus for both.

The PA genes of all H9N2 viruses (green; Figure 4) clustered with those of Bangladeshi H9N2 viruses isolated from 2011 to 2016. Surprisingly, PA genes of HPAI H5N1 viruses isolated from LPMs clustered in two distinct groups. The first group, designated as H5N1-R1, clustered with PA genes of HPAI H5N1 viruses that started to circulate in Bangladesh in June 2015, [A/duck/Bangladesh/25683/2015(H5N1)]-like viruses (red, Figure 4). The second group clustered with PA genes of Eurasian lineage, non-H9N2 LPAI viruses as a monophyletic subgroup (purple; Figure 4) sharing a common ancestor with A/duck/Bangladesh/30828/2016(H3N8). Viruses belonging to this group were first isolated from Bangladeshi LPMs in May 2017 and designated as H5N1-R2 (Figure 4). In January 2018 we isolated two H5N2 viruses, and PA genes of these two viruses are closely related to those of H5N1-R2 viruses (yellow-green; Figure 4).

**Figure 5.**
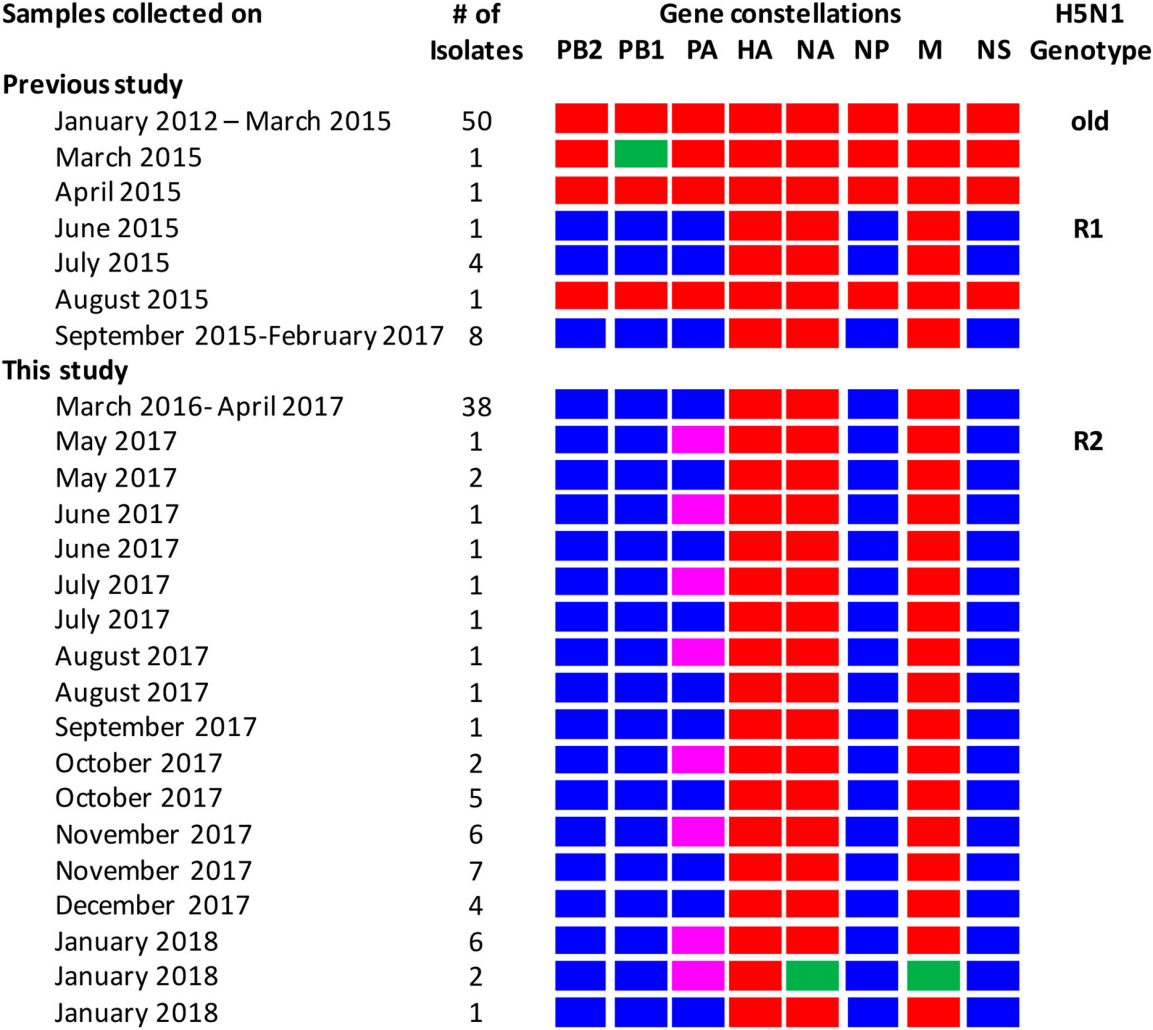
Gene constellations of HPAI H5N1 viruses isolated in Bangladeshi LPMs. Gene segments of Bangladeshi old genotype (2011–2015) HPAI H5N1-like (red), Eurasian-lineage LPAI-like (blue and purple, which belong to two distinct groups in the phylogenetic tree [Figure 4]), and H9N2-like (green) viruses are depicted for each isolated virus. On the basis of gene constellations, Bangladeshi HPAI H5N1 viruses are divided in three major groups: R1, R2, and old genotype. Note that in January 2018, we identified two H5N2 viruses with gene segments possibly derived from Bangladeshi H5N1-R1 (HA, PB2, PB1, NP, and NS), H5N1-R2 (PA), and H9N2 (NA and M) viruses.

Phylogenetic analysis of other internal genes revealed that PB2, PB1, NP, M, and NS genes of H5N1-R2 viruses originated from Bangladeshi H5N1-R1 viruses (purple; Figure S4A–E). Together, these results indicate the emergence of a new genotype of HPAI H5N1 viruses (H5N1-R2) that is possibly a reassortant of H5N1-R1 and A/duck/Bangladesh/30828/2016(H3N8)-like LPAI viruses. We first isolated this H5N1-R2 virus from samples collected from a Dhaka LPM in May 2017. Over the next few months, H5N1 viruses of both genotypes H5N1-R1 and H5N1-R2 were consistently isolated from Bangladeshi LPMs (Figure 5). In January 2018, we isolated two H5N2 viruses. Phylogenetic analysis (Figures 3, 4, S1B, and S4A–E) indicated that gene segments of these two H5N2 viruses were possibly derived from Bangladeshi H5N1-R1 (HA, PB2, PB1, NP, and NS), H5N1-R2 (PA), and H9N2 (NA and M) viruses (Figure 5).

## Discussion

Since we began conducting avian influenza surveillance in Bangladesh in 2008, multiple introductions of highly pathogenic H5N1 in Bangladesh have been reported [4–6]. Recently, we demonstrated the emergence of reassortant H5N1 viruses (H5N1-R1) with a novel genotype containing HA, NA, and M genes of HPAI H5N1 viruses previously circulating in Bangladesh (2011–2015) and five other genes of Eurasian-lineage LPAI viruses [15]. Here, we present results of our recent AIV surveillance (March 2016 – January 2018) in Bangladeshi LPMs. We found that until April 2017, H5N1 viruses exclusively belonged to H5N1-R1 genotype clade 2.3.2.1a. However, in May 2017, we identified another reassortant H5N1 virus (H5N1-R2), also of clade 2.3.2.1a, in which the PA gene segment of H5N1-R1 was replaced by that of another Eurasian-lineage LPAI. Phylogenetic analysis revealed that the PA gene of H5N1-R2 is closely related to A/duck/Bangladesh/30828/2016(H3N8), which was isolated in September 2016 from Bangladeshi LPMs. Although both reassortant H5N1-R1 and H5N1-R2 currently co-circulate in Bangladeshi LPMs, whether they will continue to co-circulate in the coming years remains to be discovered.

In HI assays, a group of H5N1 viruses exhibited a distinct antigenic profile. Ferret antisera against A/duck/Bangladesh/19097/2013(H5N1) yielded an HI titre less than 20 to these viruses compared with a homologous HI titre of 320 (Table S1), which indicated considerable antigenic drift. Interestingly, the phylogenetic tree revealed that HA genes of these low reacting viruses clustered together (Figure 3). Molecular analysis indicating amino acid changes D154N and T156A/E (Table S2) in HA1 antigenic site D [18,19] may correlate to this antigenic drift. In these HAs D154N mutation is always accompanied by T156A/E mutation, which excludes the introduction of a potential N-glycosylation site.

In January 2018, we isolated two H5N2 viruses. Phylogenetic analysis (Figures 3, 4, S3, and S4) indicated that gene segments of these H5N2 viruses derived from reassortment of Bangladeshi H5N1-R1 (PB2, PB1, HA, NP, and NS), H5N1-R2 (PA), and H9N2 (NA and M) viruses. Although reassortant H5N1 viruses having the M or PB1 gene from H9N2 viruses have been isolated from Bangladeshi LPMs before, these viruses did not establish and quickly disappeared. Along with other subtypes, Eurasian-lineage LPAI H5N2 viruses have also been identified in Bangladesh [6]. However, for the first time in Bangladesh, we have identified HPAI H5N2 viruses having NA and M genes from Bangladeshi H9N2 viruses. It will be interesting to see whether these newly identified HPAI H5N2 viruses continue to circulate or seemingly disappear like other reassortants of H5N1 and H9N2 viruses in Bangladesh.

During our surveillance period (March 2016 –January 2018), five H3N1, two H3N2, three H3N8, one H4N6, and one H6N2 LPAI viruses (along with some mixed-subtype viruses) have been isolated from Bangladeshi LPMs (Figure 2(A–C)). Phylogenetic analyses revealed that all gene segments of these viruses belong to the Eurasian lineage and also strongly suggested continuous reassortment and long-distance movement in LPAI viruses [15,20]. Some gene segments from LPAI viruses isolated from LPMs during 2016–2017 were closely related to those of LPAI viruses isolated from ducks in free-range farms and wild birds in Tanguar haor, Bangladesh, where ducks have frequent contact with migratory birds. For example, the PA genes of A/duck/Bangladesh/31019/2016(H3N2) and A/duck/Bangladesh/33676/2017(H4N6) clustered with those of viruses isolated from Tanguar haor in 2015 (grey arrows, Figure 4) [15]. This finding suggests movement of LPAI viruses from Tanguar haor-like ecosystems to LPMs. In the wet season (June to October) many of the free-range ducks from the Tanguar haor wetlands are sold off through the live poultry market system. Since these free-range ducks have shared the same wetlands and have scavenged food together with migratory waterfowl, they provide a direct link for transmitting influenza viruses from migratory waterfowl to domestic poultry in the live poultry markets, and potentially to humans. Thus, the migratory birds in the Central Asian flyway and the domestic ducks in Tanguar haor-like wetlands serve as vectors for the reassortment of influenza A viruses. Our longitudinal surveillance for avian influenza in Bangladesh is revealing a dynamic ecosystem characterized by novel reassortant generation, selective sweeps, and co-circulation. Understanding the factors that influence these phenomena should provide the best options for intervention.

## Supplementary Material

Supplemental Material
