# Supplementary material for: Continuing evolution of highly pathogenic H5N1 viruses in Bangladeshi live poultry markets
Source: Emerg Microbes Infect. 2019 Apr 24;8(1):650–61. doi: 10.1080/22221751.2019.1605845 (PMC6493222; doi:10.1080/22221751.2019.1605845)
Supplement: Supplemental Material [file TEMI_A_1605845_SM2477.zip › Table_S1_SB_12_03_18.docx]

**Table S1. Antigenic analysis of H5N1 influenza A viruses from Bangladesh by the hemagglutination inhibition assay**

|  |  |  |  |  |  |  |  |  |
| --- | --- | --- | --- | --- | --- | --- | --- | --- |
|  |  | **αH5N1 (post-infection ferret antisera)** | | | | | |  |
|  |  |  |  |  |  |  |  |  |
|  |  |  |  |  |  |  |  |  |
| **H5N1 antigen** | **Clade** | **αCM/HK**  **/5052** | **αHubei**  **/1** | **αDk/Bd**  **/19097** | **αBS/HK**  **/1161** | **αHK**  **/6841** | **αDk/VN**  **/NCVD-1153** |  |
|  |  |  |  |  |  |  |  |  |
|  |  |  |  |  |  |  |  |  |
| **Reference antigen** |  |  |  |  |  |  |  |  |
| rg-A/common magpie/Hong Kong/5052/2007 | 2.3.2.1 | **320** | 160 | 160 | 640 | 320 | 160 |  |
| rg-A/Hubei/1/2010 | 2.3.2.1a | 160 | **640** | 320 | 320 | 640 | 320 |  |
| A/duck/Bangladesh/19097/2013 | 2.3.2.1a | 80 | 320 | **320** | 320 | 320 | 320 |  |
| rg-A/barn swallow/Hong Kong/1161/2010 | 2.3.2.1b | 640 | 320 | 160 | **640** | 320 | 160 |  |
| A/Hong Kong/6841/2010 | 2.3.2.1c | 80 | 160 | 160 | 320 | **320** | 160 |  |
| rg-A/duck/Viet Nam/NCVD-1584/2012 | 2.3.2.1c | 160 | 320 | 320 | 640 | 320 | **640** |  |
| **Test antigen** |  |  |  |  |  |  |  |  |
| A/duck/Bangladesh/30057/2016 | 2.3.2.1a | 320 | 160 | 160 | 640 | 160 | 640 |  |
| A/duck/Bangladesh/30682/2016 | 2.3.2.1a | 160 | 320 | 160 | 320 | 160 | 160 |  |
| A/duck/Bangladesh/30815/2016 | 2.3.2.1a | 20 | 80 | 160 | 10 | 80 | 160 |  |
| A/duck/Bangladesh/30884/2016 | 2.3.2.1a | 80 | 80 | 160 | 640 | 160 | 160 |  |
| A/duck/Bangladesh/31023/2016 | 2.3.2.1a | 80 | 80 | 160 | 320 | 160 | 40 |  |
| A/duck/Bangladesh/31096/2016 | 2.3.2.1a | 40 | 40 | 160 | 320 | 160 | 80 |  |
| A/mallard/Bangladesh/31549/2016 | 2.3.2.1a | 80 | 160 | 160 | 320 | 320 | 160 |  |
| A/duck/Bangladesh/32502/2017 | 2.3.2.1a | 40 | 40 | 80 | 160 | 80 | 80 |  |
| A/quail/Bangladesh/32942/2017 | 2.3.2.1a | 80 | 160 | 160 | 320 | 160 | 160 |  |
| A/chicken/Bangladesh/33167/2017 | 2.3.2.1a | 40 | 160 | 160 | 160 | 160 | 320 |  |
| A/duck/Bangladesh/33287/2017 | 2.3.2.1a | 40 | 160 | 160 | 160 | 160 | 320 |  |
| A/duck/Bangladesh/33415/2017 | 2.3.2.1a | 80 | 160 | 160 | 320 | 320 | 320 |  |
| A/duck/Bangladesh/33772/2017 | 2.3.2.1a | 80 | 160 | 160 | 320 | 320 | 640 |  |
| A/duck/Bangladesh/33841/2017 | 2.3.2.1a | 80 | 320 | 160 | 320 | 320 | 320 |  |
| A/duck/Bangladesh/34036/2017 | 2.3.2.1a | 80 | 160 | 160 | 320 | 320 | 320 |  |
| A/duck/Bangladesh/34105/2017 | 2.3.2.1a | 80 | 160 | 160 | 320 | 160 | 160 |  |
| A/duck/Bangladesh/34285/2017 | 2.3.2.1a | 80 | 80 | 80 | 160 | 160 | 80 |  |
| A/duck/Bangladesh/34743/2018 | 2.3.2.1a | 80 | 160 | 160 | 160 | 160 | 160 |  |
|  |  |  |  |  |  |  |  |  |
| A/duck/Bangladesh/33775/2017 | 2.3.2.1a | 20 | 10 | 20 | 80 | 40 | 40 |  |
| A/duck/Bangladesh/33892/2017 | 2.3.2.1a | <10 | 10 | 20 | <10 | 40 | 40 |  |
| A/duck/Bangladesh/34035/2017 | 2.3.2.1a | 40 | 20 | 20 | 160 | 80 | 40 |  |
| A/duck/Bangladesh/34100/2017 | 2.3.2.1a | 10 | 10 | 20 | 10 | 40 | 20 |  |
| A/duck/Bangladesh/34102/2017 | 2.3.2.1a | <10 | <10 | 10 | <10 | 20 | 10 |  |
| A/quail/Bangladesh/34292/2017 | 2.3.2.1a | <10 | <10 | 10 | <10 | 10 | 10 |  |
| A/duck/Bangladesh/34590/2018 | 2.3.2.1a | 40 | 10 | 10 | 80 | 40 | 20 |  |
| A/chicken/Bangladesh/34722/2018* | 2.3.2.1a | 160 | 40 | 10 | 640 | 160 | 80 |  |

Abbreviations: Bd, Bangladesh; BS, barn swallow; CM, common magpie; Dk, duck; HK, Hong Kong; VN, Viet Nam; rg, reverse genetic. *The only H5N2 virus in this table. Titers are expressed as the reciprocal of the highest dilution of the last dilution that completely inhibited hemagglutination of 0.5% chicken erythrocytes. Boldface/underline indicates homologous serum.
