# Supplementary material for: Continuing evolution of highly pathogenic H5N1 viruses in Bangladeshi live poultry markets
Source: Emerg Microbes Infect. 2019 Apr 24;8(1):650–61. doi: 10.1080/22221751.2019.1605845 (PMC6493222; doi:10.1080/22221751.2019.1605845)
Supplement: Supplemental Material [file TEMI_A_1605845_SM2477.zip › Table_S3_SB_12_03_18.docx]

**Table S3. Antigenic analysis of H9N2 influenza A viruses from Bangladesh by the hemagglutination assay**

|  |  |  |  |  |  |  |  |  |  |  |
| --- | --- | --- | --- | --- | --- | --- | --- | --- | --- | --- |
|  |  |  | **αH9N2 (postinfection ferret antisera)** | | | | | | |  |
|  |  |  |  |  |  |  |  |  |  |  |
|  |  |  |  |  |  |  |  |  |  |  |
| **H9N2 antigen** | **Lineage** |  | **αHK**  **/1073** | **αHK**  **/33982** | **αBd**  **/0994** | **αQu/Bd**  **/19462** | **αCk/Bd**  **/21940** | **αCk/HK**  **/G9** | **αHK**  **/308** |  |
|  |  |  |  |  |  |  |  |  |  |  |
|  |  |  |  |  |  |  |  |  |  |  |
| **Reference antigen** |  |  |  |  |  |  |  |  |  |  |
| A/Hong Kong/1073/97 | G1 |  | **160** | 160 | 10 | 10 | <10 | 20 | <10 |  |
| rg-A/Hong Kong/33982/2009-PR8 | G1 |  | 80 | **1280** | 20 | 10 | <10 | 20 | 10 |  |
| A/Bangladesh/0994/2011 | G1 |  | 40 | 80 | **2560** | 640 | 640 | 160 | 20 |  |
| A/quail/Bangladesh/19462/2013 | G1 |  | 40 | 20 | 160 | **640** | 20 | 160 | 10 |  |
| A/chicken/Bangladesh/21940/2014 | G1 |  | 40 | <10 | 320 | 320 | **640** | 160 | 10 |  |
| A/chicken/Hong Kong/G9/97 | Y280/G9 |  | 20 | <10 | 80 | 80 | 80 | **320** | 10 |  |
| rg-A/Hong Kong/308/2014-PR8 | Y280/G9 |  | 20 | 20 | 20 | 40 | 400 | 40 | **1280** |  |
| **Test antigen** |  |  |  |  |  |  |  |  |  |  |
| A/chicken/Bangladesh /30030/2016 | G1 |  | 80 | 20 | 1280 | 320 | 640 | 160 | 40 |  |
| A/chicken/Bangladesh /30861/2016 | G1 |  | 40 | 20 | 640 | 640 | 320 | 80 | 10 |  |
| A/chicken/Bangladesh/31624/2016 | G1 |  | 80 | 80 | 640 | 320 | 160 | 320 | 20 |  |
| A/chicken/Bangladesh/32287/2017 | G1 |  | 80 | 80 | 1280 | 1280 | 320 | 160 | 10 |  |
| A/chicken/Bangladesh/32390/2017 | G1 |  | 40 | 40 | 640 | 320 | 640 | 160 | 10 |  |
| A/chicken/Bangladesh/32958/2017 | G1 |  | 40 | 80 | 2560 | 640 | 320 | 320 | 20 |  |
| A/chicken/Bangladesh/33107/2017 | G1 |  | 40 | 40 | 2560 | 320 | 320 | 160 | <10 |  |
| A/duck/Bangladesh/33138/2017 | G1 |  | 40 | 40 | 2560 | 640 | 640 | 320 | <10 |  |
| A/chicken/Bangladesh/33386/2017 | G1 |  | 80 | 80 | 5120 | 640 | 1280 | 320 | <10 |  |
| A/chicken/Bangladesh/33645/2017 | G1 |  | 80 | 40 | 2560 | 640 | 640 | 320 | <10 |  |
| A/chicken/Bangladesh/34004/2017 | G1 |  | 80 | 80 | 2560 | 640 | NT | 320 | 10 |  |
| A/chicken/Bangladesh/34075/2017 | G1 |  | 40 | 40 | 1280 | 320 | NT | 160 | 10 |  |
| A/chicken/Bangladesh/34322/2017 | G1 |  | 40 | 40 | 1280 | 320 | NT | 160 | 10 |  |
| A/chicken/Bangladesh/34574/2018 | G1 |  | 80 | 40 | 640 | 320 | NT | 160 | 10 |  |
| A/chicken/Bangladesh/34637/2018 | G1 |  | 80 | 160 | 5120 | 1280 | NT | 320 | 20 |  |
|  |  |  |  |  |  |  |  |  |  |  |
| A/quail/Bangladesh/29997/2016 | G1 |  | 40 | 40 | 80 | 320 | 40 | 40 | 10 |  |
| A/quail/Bangladesh /30637/2016 | G1 |  | 20 | 20 | 40 | 160 | 20 | 80 | <10 |  |
| A/quail/Bangladesh /31043/2016 | G1 |  | 40 | 20 | 10 | 640 | 20 | 40 | <10 |  |
| A/quail/Bangladesh/32525/2017 | G1 |  | 80 | 80 | 5120 | 1280 | 1280 | 320 | 20 |  |
| A/quail/Bangladesh/32935/2017 | G1 |  | 80 | 80 | 5120 | 640 | 640 | 320 | 20 |  |
| A/quail/Bangladesh/33376/2017 | G1 |  | 40 | 80 | 80 | 160 | <10 | 40 | <10 |  |
| A/quail/Bangladesh/34042/2017 | G1 |  | 40 | 80 | 80 | 320 | NT | 40 | 10 |  |
| A/quail/Bangladesh/34615/2018 | G1 |  | 40 | 80 | 80 | 160 | NT | 40 | <10 |  |
|  |  |  |  |  |  |  |  |  |  |  |

Abbreviations: Bd, Bangladesh; Ck, chicken; HK, Hong Kong; Qu, quail.

Titers are expressed as the reciprocal of the highest dilution of the last dilution that completely inhibited hemagglutination of 0.5% chicken erythrocytes. Boldface with underline indicates homologous serum.
